# Efficient generation of thymic epithelium from induced pluripotent stem cells that prolongs allograft survival

**DOI:** 10.1038/s41598-019-57088-1

**Published:** 2020-01-14

**Authors:** Ryo Otsuka, Haruka Wada, Hyuma Tsuji, Airi Sasaki, Tomoki Murata, Mizuho Itoh, Muhammad Baghdadi, Ken-ichiro Seino

**Affiliations:** Institute for Genetic Medicine, Hokkaido University, Kita-15, Nishi-7, Sapporo, 060-0815 Japan

**Keywords:** Stem-cell differentiation, Allotransplantation

## Abstract

The thymus plays a significant role in establishing immunological self-tolerance. Previous studies have revealed that host immune reaction to allogeneic transplants could be regulated by thymus transplantation. However, physiological thymus involution hinders the clinical application of these insights. Here, we report an efficient generation of thymic epithelial-like tissue derived from induced pluripotent stem cells (iPSCs) and its potential to regulate immune reaction in allogeneic transplantation. We established an iPSC line which constitutively expresses mouse *Foxn1* gene and examined the effect of its expression during *in vitro* differentiation of thymic epithelial cells (TECs). We found that *Foxn1* expression enhances the differentiation induction of cells expressing TEC-related cell surface molecules along with upregulation of endogenous *Foxn1*. iPSC-derived TECs (iPSC-TECs) generated T cells in nude recipient mice after renal subcapsular transplantation. Moreover, iPSC-TEC transplantation to immuno-competent recipients significantly prolonged the survival of allogeneic skin. Our study provides a novel concept for allogeneic transplantation in the setting of regenerative medicine.

## Introduction

The thymus is a crucial lymphoid organ for T-cell generation. During intrathymic development, T cells produce a vast receptor repertoire for defending individuals from invasive microorganisms or eliminating *de novo* malignant neoplasms. However, repertoire expansion also provides a potential cost for self-reactivity, which leads to autoimmune disorders. To prevent the release of auto-reactive T cells from the thymus, immature T cells encounter thymic antigen presenting cells (APCs), which express a wide spectrum of self-antigen-derived peptides combined with major histocompatibility complex (MHC) molecules. This process allows elimination of auto-reactive T cells and also induces regulatory T cells^1^.

Given that the thymus functions as a site of T-cell tolerance establishment, previous research attempted thymus transplantation to prevent immunological rejection in experimental transplantation models^2–5^. Immune rejection is mainly mediated by T cells, and eliminating T cells themselves or preventing their activation contributes to prolonged survival of transplanted organs. Indeed, most currently available commercial immunosuppressants target T cells and inhibit their activation^6^.

There are several subsets of APCs in the thymus, such as dendritic cells, macrophages, or thymic epithelial cells (TECs)^7^. However, thymic lobes transplanted without haematopoietic cells, but containing epithelial structure, tolerizes the host immune system to the thymus donor mouse strain^2,3^. These results suggest that intrathymic haematopoietic APCs are not necessary for establishing donor-specific unresponsiveness. Therefore, transplantation of thymic epithelium might be beneficial for preventing immune rejection. However, despite its therapeutic potential, thymus grafting to organ transplantation recipients has not reached clinical settings. This would be because of physiological involution of the thymus. The thymus has its maximum size and potential for T cell generation during childhood; its function decreases with aging. The degenerated thymus is typified by a reduced number of T cells and TECs; however, adipose tissues are broadly observed^8^. Thus, considering that organ donors are usually adult individuals, physiological involution results in decreased thymus availability from organ donors. Even though it is possible to graft an aged thymus, there would be less ability to tolerize the recipient immune system.

Pluripotent stem cells such as embryonic stem cells (ESCs) and iPSCs are expected to be an alternative source of grafts for transplantation. Recently, it has been reported that mouse and human ESCs can be induced to differentiate into thymic epithelial-like cells *in vitro*^9–12^; however, these previous reports also showed difficulties with efficient TEC derivation. Moreover, application of these pluripotent stem cell-derived TECs to induce donor-specific tolerance has not been investigated.

Here, we report an alternative way to induce iPSC-derived TECs through a stepwise *in vitro* differentiation protocol along with *Foxn1* gene transduction. *Foxn1* transduction significantly enhanced TEC induction efficiency with upregulation of TEC-related marker genes. Furthermore, our results raise the possibility that iPSC-derived TECs transplanted into allogeneic recipients contribute to prolonged survival of the transplants whose MHC is identical to iPSC-TECs.

## Results

### Conditioning thymic epithelial cell differentiation

**Figure 1:**
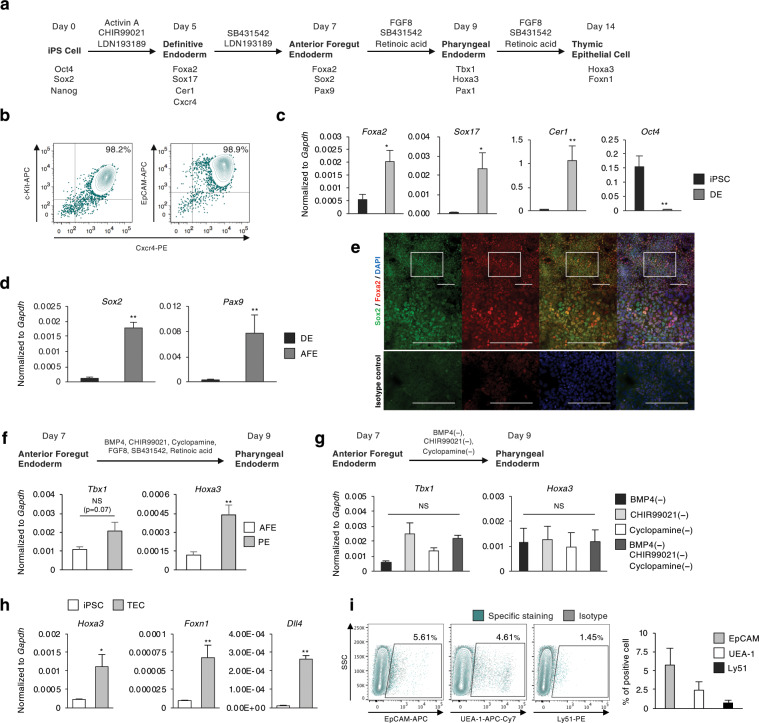
Generation of iPSC-TECs. (**a**) Schematic showing the differentiation protocol of thymic epithelial cells. (**b**) Definitive endoderm marker expression on day 5 of differentiation. Plots show representative flow cytometric analysis. (**c**) Expression of *Foxa2*, *Sox17*, *Cer1*, and *Oct4* on day 5 of differentiation. Definitive endoderm markers (biological replicates: *Foxa2*, n = 8; *Sox17*, n = 7; *Cer1*, n = 6) and Pluripotency gene (*Oct4*, n = 8). (**d**) RT-qPCR analysis of anterior foregut marker gene expression on day 7 of differentiation. Definitive endoderm cells were treated with SB431542 and LDN193189 for 2 days (n = 3, biological replicates). (**e**) Double immunostaining for Foxa2 (green) and Sox2 (red). Nuclei were counterstained with DAPI (blue). Lower panels show enlargement of insets in upper panels. Scale bars represent 200 μm. (**f**) RT-qPCR analysis of *Tbx1* and *Hoxa3* (n = 6 and n = 7, respectively, biological replicates) as pharyngeal pouch endoderm marker genes on day 9. (**g**) Optimizing pharyngeal endoderm differentiation conditions. Each bar represents culture condition without the indicated factors. NS, not significant, Tukey’s multiple comparison test (n = 3, biological replicates). (**h**) RT-qPCR analysis for TEC-related genes (*Hoxa3*, *Foxn1*, and *Dll4*) on day 14 of differentiation (n = 3, biological replicates). (**i**) Representative plots of cell surface analysis of TEC-related marker molecules and average induction rate of each marker-positive cell (n = 3, biological replicates). Plots show live cell (DAPI^−^) gated population. *p < 0.05, **p < 0.01, two-tailed Student’s t-test. DE: definitive endoderm; AFE: anterior foregut endoderm; PE: pharyngeal endoderm; TEC: thymic epithelial cell.

The thymus is of endodermal origin, sharing its ancestor with respiratory or gastric organs such as the lung, liver, or pancreas^13^. We first focused on constructing a step-by-step protocol for induction of TECs through definitive endoderm (DE), anterior foregut endoderm (AFE), and pharyngeal endoderm (PE) (Fig. 1a). DE is known to be induced by a high concentration of Activin and is defined by cell surface expression of Cxcr4, c-Kit, and EpCAM^14^. We established a protocol for DE induction by modifying several induction methods to optimize them for our iPS cell line (Supplementary Figs. 1–4). Flow cytometric analysis revealed highly overlapped expression of these marker molecules, suggesting efficient (c-Kit^+^Cxcr4^+^ cells were 86.7% ± 3.25) DE induction (Fig. 1b). Additionally, upregulation of DE marker genes, defined by quantitative PCR (qPCR), also indicated appropriate differentiation (Fig. 1c). We also examined Foxa2 protein expression and found it to be primarily localized to the nucleus, whereas protein expression of Sox2, one of the key factors responsible for pluripotency, was not detected (Supplementary Fig. 5). These results are consistent with the estimated efficiency determined by flow cytometry (Fig. 1b).

The thymus arises from an anterior portion of developing endoderm, AFE^13^. The early anterior-posterior formation is regulated by bone morphogenetic protein (BMP), fibroblast growth factor (FGF), and Wnt/β-catenin signalling. Previously, screening multiple combinations of signal agonists and antagonists revealed that DE cells could be anteriorized by simultaneous inhibition of transforming growth factor (TGF) and BMP signaling^15^. By culturing DE cells in the presence of these signal inhibitors, i.e., SB431542 and LDN193189, anterior marker genes were significantly upregulated, and immunofluorescence imaging showed merged expression of Sox2 with Foxa2 (Fig. 1d,e). Importantly, suppressed Sox2 expression at the DE stage was restored after the anteriorization process. To induce pharyngeal endoderm, anterior foregut endoderm cells were exposed to 6 factors to stimulate BMP, FGF, Wnt/β-catenin, and retinoic acid (RA) signalling whilst under combinatory inhibition of TGF and the sonic hedgehog signalling pathway^10,15^. This induction step efficiently induced pharyngeal pouch marker *Tbx1* and *Hoxa3* (Fig. 1f). Because TECs are known to develop from the *Hoxa3*-expressing region in the 3rd pharyngeal pouch (3rd p.p.)^16,17^, we focused on optimizing culture conditions by using *Hoxa3* as a guide for sufficient 3rd p.p. induction. We identified that single or combinatory withdrawal of BMP4, CHIR99021, or cyclopamine from the induction conditions did not affect *Hoxa3* and *Tbx1* expression on day 9 (Fig. 1g). However, subtracting FGF8, SB431542, or RA resulted in reduced expression of *Tbx1* and *Hoxa3* (data not shown).

We found that thymic specification can be carried out by continuous exposure to FGF8, SB431542, and RA until day 14 of differentiation (morphological changes are shown in Supplementary Fig. 5). Physiological TEC differentiation is dependent on region-specific expression of forkhead box protein, Foxn1^18^. Hence, we employed *Foxn1* as a key differentiation marker and quantified its expression on day 14. A small but significant increase in *Foxn1* expression indicated the existence of a TEC population in the obtained cells (Fig. 1h). We also analysed marker molecules on the cell surface by flow cytometry. TECs are categorized as an epithelial cell subset, and approximately 5% of obtained cells expressed epithelial cell adhesion molecule (EpCAM). TECs are further classified into two subsets based on their surface expression of Ly51 and UEA-1, which are expressed by cortical and medullary TECs, respectively^19^. Flow cytometry analysis revealed minimal levels of these populations (Fig. 1i). Bilateral cross-talk between developing thymocytes and TEC progenitors is an essential event during mature TEC development^1^. However, it is indicated by cell surface analysis that the *in vitro* culture condition promoted terminal differentiation without receiving signals from T cells. Notably, gene expression of delta-like 4 ligand (*Dll4*) was also determined by qPCR, and the results suggest the possibility of T-cell differentiation (Fig. 1h).

### Transduction of exogenous Foxn1 gene promotes iPSC-TEC differentiation

**Figure 2:**
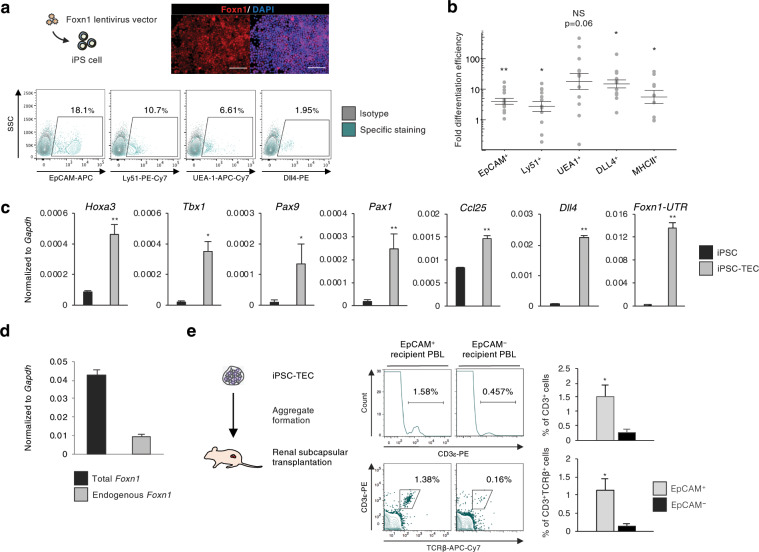
*Foxn1* gene transduction and phenotypic/functional characterization of iPSC-TECs. (**a**) TEC differentiation with Foxn1-expressing mouse iPSC. Fluorescent images show double staining for Foxn1 (red) and the nucleus (blue). Scale bars represent 100 μm. Flow cytometric plots show representative flow cytometric analysis of TEC-related marker molecules on day 14 of differentiation. (**b**) Fold induction efficiency of marker-expressing cells compared with normal (not transduced with *Foxn1*) iPSCs. (**c**) Expression of *Hoxa3*, *Tbx1*, *Pax9*, *Pax1*, *Ccl25*, *Dll4*, and *Foxn1*-UTR on day 14 of differentiation. *Foxn1*-UTR expression was analysed by primers specific for 3′-UTR regions of *Foxn1* mRNA. (n = 8, biological replicates). (**d**) Expression of “Total” and “Endogenous” Foxn1 on day 14 of differentiation (n = 4, biological replicates). (**e**) Schematic of Foxn1-iPSC-derived transplantation of TEC into nude recipients (left). iPSC-TECs (EpCAM^+^, Ly51^+^, UEA-1^+^) were sorted, and aggregates (1 × 10^5^ iPSC-TECs were mixed with 3 × 10^4^ MEFs) were transplanted into nude mice. Peripheral blood analysis of nude mice 6 weeks after transplantation. Nude mice received iPSC-TEC aggregates without DN1 thymocytes. Plots show live cell (DAPI^−^) and recipient blood cell (CD45.2^+^) gated populations (right). (*p < 0.05, **p < 0.01, Student’s t-test).

*Foxn1* is known as a key transcription factor of thymus morphogenesis. Loss-of-function mutation of *Foxn1* results in athymia and nude phenotype because of defects in hair follicle formation^20^. We next sought to address whether this developmental control gene would also affect *in vitro* generation of TECs. To evaluate this possibility, we generated a mouse iPS cell line carrying an exogenous gene construct that encodes mouse *Foxn1* under the human EF1α promoter. We did not observe any change in cell growth activity or toxicity with *Foxn1*-transduced iPSCs (data not shown). TEC induction was carried out by following our induction protocol (Figs. 1a, 2a). Flow cytometric analysis revealed that inducing *Foxn1* expression during *in vitro* differentiation had a promotive effect on TEC induction (Fig. 2b). Comparing the differentiation results showed the significantly enhanced induction efficiency of TEC-related molecule expressing cells (Fig. 2b).

We also analysed developmental genes such as *Hoxa3*, *Tbx1*, and *Pax9*, which showed a significant increase through differentiation induction (Fig. 2c). Additionally, *Ccl25* and *Dll4* were also upregulated (Fig. 2c). As it is known that the molecular expression of Ccl25 and Dll4 can be influenced by, but not dependent on^21–23^, Foxn1, there was a possibility that the upregulation of these genes was not caused by differentiation induction but rather induced by exogenous *Foxn1* expression. We also analysed *Foxn1* expression by a specific primer pair targeting the 3′-UTR of *Foxn1*^24^, which enabled us to distinguish exogenous from endogenous *Foxn1*. Although the influence of exogenous *Foxn1* cannot be excluded, significantly upregulated expression of “endogenous *Foxn1*” indicated that the iPSC-derived cultured cells accurately differentiated into thymic epithelial lineage (Fig. 2c). We also evaluated the expression level of total (endogenous + exogenous) *Foxn1* and found that estimated exogenous (total – endogenous) *Foxn1* expression level was higher than that of endogenous *Foxn1*. This might due to sustained strong expression of transduced *Foxn1* gene.

### iPSC-TECs generate T cells in nude mice recipients

We then examined the functional capacity of iPSC-TECs by transplantation of these cells with immature T-cell progenitors into athymic nude mice (Supplementary Fig. 6a,b). Flow cytometric analysis revealed physiological-like development of CD4 or CD8 single-positive cells derived from co-cultured DN1 cells (Supplementary Fig. 6c). Furthermore, when the differentiated cells were sorted by their expression of EpCAM, we observed a 10-fold increase in the proportion of T cells in peripheral blood from EpCAM^+^ cell recipient nude mice compared with EpCAM^−^ cell recipients (Fig. 2e). Consistent with this, TCRVβ repertoire expression analysis showed a variety of TCRVβ generation in recipient T cells (Supplementary Fig. 7). These results demonstrated that exogenously expressed *Foxn1* exerted a promotive effect on *in vitro* generation of iPSC-TECs that reconstituted host T cells with a wide TCR repertoire range.

### iPSC-TECs contribute to skin graft survival in allogeneic recipients

**Figure 3:**
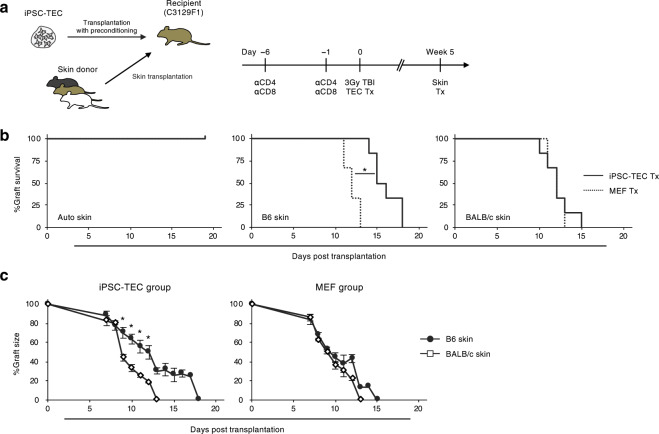
Immune-regulatory functions of iPSC-TECs. (**a**) Schematic overview of iPSC-TEC and skin transplantation. Timeline shows the procedure of recipient preconditioning and skin transplantation. (**b**) Kaplan-Meier curves for each skin graft are shown with p-value (B6 skin). Solid line, survival of each skin graft transplanted to iPSC-TEC recipients (n = 6). Dotted line graft survival in MEF (control cell) aggregate recipients (n = 6). (*p < 0.05, generalized Wilcoxon test) (**c**) Percent graft size of B6 and BALB/c skin after transplantation with error bars on each day of size measurement. (left) iPSC-TEC aggregates pre-transplanted group, (right) MEF aggregates pre-transplanted group. Statistical significance was evaluated when n ≥ 3 at each time point. (*p < 0.05, two-tailed Student’s t-test).

MHC-homozygous iPSCs are expected to be less immunogenic when transplanted into MHC-homozygous recipients, and the MHC-homozygous iPSCs are banked for the therapeutic use of iPSC-derived grafts^25^. Thereafter, we evaluated the effect of iPSC-TECs on allograft survival, in which we used skins instead of iPSC-derived therapeutic cells. C3129F1 mice (recipients) received renal subcapsular transplantation of C57BL/6 (B6) iPSC-TEC aggregates following preconditioning of anti-CD4/CD8 antibodies and total body irradiation (Fig. 3a). For control group mice, B6 MEF aggregates were transplanted to assess the possibility of B6 cell influence on recipient “anti-B6” immune responsiveness. Anti-T cell antibody treatment efficiently depleted peripheral CD4^+^ or CD8^+^ T cells, and we observed peripheral T cell recovery 5 weeks after the aggregate transplantation (Supplementary Fig. 8). Despite the heterozygous MHC haplotype (H-2^b/k^) in C3129F1 mice and homozygous haplotype in B6 mice (H-2^b^), skin grafting from B6 to control C3129F1 caused rapid rejection and resulted in graft loss. In contrast, recipients of iPSC-TEC aggregates showed significantly prolonged survival of B6 skin (Fig. 3b centre panel; median survival time (MST) of 15.5 days). Moreover, skins from third-party BALB/c (H-2^d^) were rejected independently of pre-transplanted subrenal capsule iPSC-TEC aggregates (Fig. 3b,c; MST = 12 days), and auto skin grafts showed complete engraftment (Fig. 3b; MST > 20 days). The recipients of MEF aggregates rapidly rejected B6 (MST = 12 days) and BALB/c (MST = 12 days) skin graft. These results suggest that iPSC-TEC transplantation reconstitutes the host immune system and specifically extends the survival of allografts from the same donor strain.

## Discussion

In this study, we have demonstrated that mouse iPSCs, integrated with exogenous *Foxn1* gene, efficiently differentiate into thymic epithelial cells, and transplantation of *in vitro* generated thymic aggregates induces prolonged survival of transplanted grafts. Although skin allograft rejection and antigen-specific T-cell clone deletion were previously investigated in PSC-TEC recipients^10^, our study demonstrates for the first time that iPSC-TECs contribute to allograft survival in immunocompetent mice.

Previous studies aiming to generate thymic epithelium from PSCs showed successful differentiation from PSCs to TECs^9–11,26^. However, the efficiency of the TEC differentiation was generally low, and Su *et al*. utilized a recombinant transcription factor, HOXA3, and FOXN1^12^. They demonstrated promotion of the differentiation efficiency and functional T-cell generation in nude mice. We induced exogenous *Foxn1* expression during iPSC differentiation that promoted *in vitro* TEC generation. This result suggests that *Foxn1* influences the fate of differentiating cells. Consistent with this, Bredenkamp *et al*. showed that enforced expression of *Foxn1* in mouse fibroblasts promotes TEC-like phenotype acquisition^24^. The induction efficiency of target cells varies depending on cell line, and protocols often need to be optimized for each cell line. Although it is difficult to exactly compare the utility of the TEC induction protocol with another mouse iPSC-TEC study^27^, enforcement of Foxn1 expression in iPSC may enhance differentiation efficiency, whichever protocol is used. Future studies are needed to extend our findings.

*Foxn1* is also known to be expressed and function in hair follicles^28^. However, we excluded the possibility of hair follicle cell generation by analysing ectodermal gene expression. Moreover, these types of genes were not detected in our differentiation protocol (data not shown). Together, these results suggest that the T-cell generating function was not simply due to phenotypic acquisition but also caused by successful differentiation toward TECs. Transplanted iPSC-TECs were only sufficient to generate a small number of T cells *in vivo*. One of the possible reasons is that iPSC-TECs after isolation can survive for only a short period. The differentiation medium contains various supplements that may influence the cells’ demand for nutrients. Fine-tuning of medium supplements may be beneficial for sustained engraftment of iPSC-TECs. Another possible reason is that iPSC-TECs are not capable of efficiently recruiting immature T cells from peripheral circulation. Consistently, double-negative thymocytes mixed with iPSC-TECs differentiated into mature T cells within aggregates. iPSC-TECs expressed T-cell recruiting chemokine *Ccl25*; however, its protein level and secretion may not be effective for T-cell infiltration into iPSC-TEC aggregates.

B6 iPSC-TECs significantly prolonged the survival of skin grafts from the B6 donor, specifically, albeit to a lesser extent. It could be reasoned that there is a hierarchy in the susceptibility of different allografts to rejection, and the skin is ranked as one of the most susceptible transplants to rejection^29^. Thymus transplantation following appropriate pre-treatment reconstitutes the recipient’s T-cell immune system. To establish immune tolerance against the thymus donor, T cells developed in the donor thymus would need to be dominant. It is supposed that donor thymus-derived T cells exist as a small proportion and could not convert the T-cell immune system to donor tolerant. Regulatory T cells also play significant roles in T-cell tolerance. Even in cases where those cells were generated through iPSC-TECs, their numbers were not sufficient to regulate anti-donor response. While we treated recipient mice with a T-cell depletion antibody and total body irradiation to modify their immune system to become similar to that of nude mice, there is an involved preconditioning regimen in addition to T-cell depletion (e.g., co-stimulatory molecule blockade). In addition, anti-recipient thymus treatment is also needed to precisely balance T-cell development. Treating recipient mice with another kind of conditioning method prior to iPSC-TEC or skin transplantation would facilitate allograft survival for even longer periods.

Our research group has recently achieved the regulation of recipient immune response against PSC-derived allograft by the administration of PSC-derived immunoregulatory cells^30,31^. Although in several previous studies, thymus transplantation surmounted discordant allogeneic barrier^2–5^, this approach has still not reached the clinic. Our notion will provide a possibility for iPSCs to be an alternative source of thymus graft which is difficult to procure from a living or cadaveric donor. Further, even in PSC-based therapy, our conceptual studies would contribute to the successful development of regenerative medicine.

## Methods

### Mice

C57BL/6, BALB/c, C3H/He, 129X 1/SvJJmsSlc and BALB/c^nu/nu^ mice were purchased from Japan SLC, Inc. C3129F1 mice were obtained by mating female C3H with male 129 mice in our own colony. All animal procedures were approved by the Hokkaido University Animal Care Committee (Approval number: 17–0110) and the methods were performed in accordance with the Guide for the Care and Use of Laboratory Animals published by the National Institutes of Health.

### Cells and culture condition

The B6 mouse iPS cell line was established in our previous study^32^. iPSCs were maintained on laminin-coated culture dishes with maintenance medium consisting of Advanced-DMEM/F-12 (1:1 mixed, Thermo, SIGMA) supplemented with 0.5× Neuro Brew-21 (Miltenyi), 0.5× N2 supplement (Wako), 0.1 mM non-essential amino acids, 1 mM sodium pyruvate, 10 U/ml penicillin, 100 μg/ml streptomycin, 0.1 mM 2-mercaptoethanol (Nacalai), 0.03% L-Glutamine (Gibco), 3 μM CHIR99021 (Adooq), PD0325901 (Tocris), and recombinant human leukaemia inhibitory factor (produced in our laboratory). Cells were maintained in a 5% CO_2_/air environment at 37 °C.

### Cloning and gene transduction with lentiviral vector

Mouse *Foxn1* CDS was cloned into pLenti-EF1a-C-Myc-DDK-IRES-Puro vector (Origene), and B6 iPSCs were infected with lentivirus. Lenti-X293T cells were transfected with lentiviral vector and two packaging plasmids, pMD2.G and psPAX2 (Addgene), using polyethylene imine. Lentivirus-containing supernatants were collected and mixed with 1/3 volume of polyethylene glycol solution. The mixture was incubated at 4 °C overnight and centrifuged at 1500 × *g* for 30 min. After removing the supernatant, pellets were centrifuged again at 1500 × *g* for 5 min to completely remove remaining supernatant. Concentrated virus pellets were reconstituted in serum-free media and frozen at −80 °C. To generate a Foxn1-expressing iPS cell line, concentrated virus solution was added into the maintenance culture of iPS cells, and a stable cell line was established by puromycin selection.

### Thymic epithelial cell induction

Several definitive endoderm induction methods were modified and optimized for our iPSC line^15,33,34^. Briefly, the induction was performed in a serum-free differentiation medium (SFD)^34^, d0-2, d3-5, and serum-containing differentiation medium (SCD), d2-3. SFD consisted of 75% IMDM (Sigma), 25% Ham’s F-12 nutrient mixture (Sigma) with 1× Neuro Brew-21 (vitamin A+), 1× N2 supplement, 10 U/ml penicillin, 100 μg/ml streptomycin, 0.05% BSA, 0.03% L-Glutamine, 0.5 mM ascorbic acid, and 4.5 × 10^−4^ M monothioglycerol (Sigma). Neuro Brew-21 and N2 supplement were substituted with 2% fetal bovine serum (Sigma) to produce SCD. iPS cells were trypsinised and seeded on a gelatin-coated culture dish (8000 cells/cm^2^) with SFD on day 0 of induction. On day 2 of differentiation, the medium was replaced with SCD containing 50 ng/ml Activin A and 6 μM CHIR99021, GSK3 inhibitor. The medium was replaced with SFD containing 50 ng/ml Activin A and 0.1 μM LDN193189 (a BMP signal inhibitor) on day 3 and 4. Definitive endoderm cells (day 5) were confirmed by flow cytometry and qPCR analysis. Cells were anteriorized by culturing in 0.05% FBS/SFD with 10 μM SB431542, TGF signal inhibitor, and 0.1 μM LDN193189 for the following 2 days. Anterior foregut endoderm cells (day 7) were then exposed to 50 ng/ml FGF8, 10 μM SB431542, and 0.1 μM retinoic acid in 0.05% FBS/SFD for 7 days to induce TEC differentiation. Terminally differentiated TECs were detached from the culture dish by treating with accutase (Nacalai), and cell surface molecules and gene expression were analysed.

### Immunofluorescence

Immunofluorescence staining for cultured cells was performed on μ-slide 8 well (ibidi). Cells were fixed in 4% paraformaldehyde for 15 min at room temperature, washed twice in Tris-buffered saline with 0.05% tween 20 (TBS-T) and blocked 60 min with blocking solution (TBS-T containing 1% bovine serum albumin and 1% Block Ace). The fixed cells were immersed in a permeabilizing solution (0.2% Triton-X100/TBS-T) for 10 min. Primary antibodies, Foxa2, Sox2, and Foxn1, were prepared as 1:200 dilution with antibody diluent (1%BSA/TBS-T) and added overnight at 4 °C (Supplementary table 2). Cells were washed and incubated with donkey anti-rabbit IgG-AlexaFlour555, goat anti-mouse IgG-AlexaFlour488, and donkey anti-goat IgG-AlexaFlour555 for 1 h at 4 °C. Nuclei were stained with DAPI. Stained samples were visualized using fluorescent microscopy (Zeiss Observer Z1, Zeiss) and images were captured and analyzed by AxioVision software (Zeiss).

### Flow cytometry analysis and cell sorting

Flow cytometry analysis for *in vitro* cultured cells was performed by the following procedure. Cells attached to the culture dish were washed in PBS(−) and trypsinised for 5 min. The trypsin reaction was neutralized by the addition of culture medium containing 10% FBS and collected cells were centrifuged. Pellets were re-suspended and filtered before proceeding to staining (Supplementary table 2).

### Reverse-transcription and quantitative PCR

Total RNA was extracted using TriPure (Roche Diagnostics GmbH). RNA was reverse transcribed into cDNA using First Strand cDNA Synthesis Kit ReverTra Ace (Toyobo). All experiments were performed in technical duplicate with the KAPA SYBR Fast qPCR Kit (Nippon Genetics). Primer pairs are listed in Supplementary Table 1.

### Transplantation

For iPSC-TEC aggregate transplantation, sorted 1 × 10^5^ iPSC-TECs were mixed with 3 × 10^4^ MEFs and cultured overnight on an 8 μm-pore semipermeable filter in SFD medium; formed aggregates were transplanted under the kidney capsule. In experiments with DN1 thymocytes, E14 fetal thymus DN1 cells were sorted, and 1 × 10^3^ cells were mixed with 1 × 10^5^ iPSC-TECs and 2.5 × 10^4^ MEFs per aggregate. Recipient C3129F1 mice were administrated with anti-CD4 (GK1.5) and anti-CD8 (53-6.7) monoclonal antibodies containing mouse ascites on day 1 and 6 of transplantation. At least 4 weeks after iPSC-TEC or MEF aggregate transplantation, peripheral blood cells were analysed by flow cytometry to confirm T-cell reconstitution. All recipient mice, including nude mice, received 3 Gy of total body irradiation 6 h before renal subcapsular transplantation.

For skin transplantation, ear skins from the dorsal side of B6, BALB/c, and C3129F1 mice were transplanted into the dorsal thoracic wall of C3129F1 recipients as previously described^35^. Graft survival was monitored by macroscopic inspection. Recipient mice were anesthetized by intraperitoneal injection of a three-drug mixture of medetomidine (Domitor® Nippon Zenyaku Kogyo Co., Ltd.), midazolam (midazolam injection, TEVA®, Takeda Pharmaceutical Co., Ltd.), and butorphanol (Vetorphale®, Meiji Seika).

### TCR repertoire diversity analysis

TCR repertoire of recipient splenic T cells was analyzed by Anti-Mouse TCR Vβ Screening Panel (BD Bioscience). We measured the diversity of TCR Vβ repertoire by the Shannon entropy as previously described^36,37^. Entropy (H) was calculated as H ≡ Σp_*i*_log_2_p_*i*_, where p_*i*_ is the frequency of Vβ repertoire *i*.

### Statistical analyses

Statistical data were analyzed with JMP® 14 (SAS Institute Inc.) using Student’s *t*-test (unpaired, two-tailed) or Tukey’s multiple comparison test. Kaplan–Meier survival curves were analyzed using generalized Wilcoxon test. *p*-Value was considered statistically significant when <0.05.

## Supplementary information

Supplementary Information.
