# Author Correction: Efficient generation of thymic epithelium from induced pluripotent stem cells that prolongs allograft survival

**DOI:** 10.1038/s41598-021-92226-8

**Published:** 2021-06-14

**Authors:** Ryo Otsuka, Haruka Wada, Hyuma Tsuji, Airi Sasaki, Tomoki Murata, Mizuho Itoh, Muhammad Baghdadi, Ken-ichiro Seino

**Affiliations:** Institute for Genetic Medicine, Hokkaido University, Kita-15, Nishi-7, Sapporo, 060-0815 Japan

Correction to: *Scientific Reports* 10.1038/s41598-019-57088-1, published online 14 January 2020

The Supplementary Information file published with this Article contained an error, where the Supplementary Tables 1 and 2 were omitted.

The original Supplementary Information file is provided below.

As a result, the in-text citations for Supplementary Table 2 in the Methods section, under the subheadings 'Immunofluorescence’ and ‘Flow cytometry analysis and cell sorting’, were omitted.

“The fixed cells were immersed in a permeabilizing solution (0.2% Triton-X100/ TBS-T) for 10 min. Primary antibodies, Foxa2, Sox2, and Foxn1, were prepared as 1:200 dilution with antibody diluent (1%BSA/TBS-T) and added overnight at 4 °C.”

now reads:

“The fixed cells were immersed in a permeabilizing solution (0.2% Triton-X100/ TBS-T) for 10 min. Primary antibodies, Foxa2, Sox2, and Foxn1, were prepared as 1:200 dilution with antibody diluent (1%BSA/TBS-T) and added overnight at 4 °C (Supplementary table 2).”

“Pellets were re-suspended and filtered before proceeding to staining.”

now reads:

“Pellets were re-suspended and filtered before proceeding to staining (Supplementary table 2).”

The original Article and accompanying Supplementary Information file have been corrected.

## Supplementary Information

Supplementary Information.

