# Supplementary material for: Efficient generation of thymic epithelium from induced pluripotent stem cells that prolongs allograft survival
Source: Sci Rep. 2020 Jan 14;10:224. doi: 10.1038/s41598-019-57088-1 (PMC6959230; doi:10.1038/s41598-019-57088-1)
Supplement: Supplementary file 1 — Supplementary Information. [file 41598_2019_57088_MOESM1_ESM.pdf]

Article

Efficient generation of thymic epithelium from induced pluripotent stem cells that prolongs allograft survival

Ryo Otsuka[1], Haruka Wada[1], Hyuma Tsuji[1], Airi Sasaki[1], Tomoki Murata[1], Mizuho Itoh[1], Muhammad Baghdadi[1], and Ken-ichiro Seino[1]*

[1]Institute for Genetic Medicine, Hokkaido University, Kita-15, Nishi-7, Kita-ku, Sapporo, Hokkaido, 060-0815, Japan

*Correspondence to:

Ken-ichiro Seino, M.D., Ph.D.

Institute for Genetic Medicine, Hokkaido University

Kita-15, Nishi-7, Kita-ku, Sapporo, Hokkaido, 060-0815, Japan

Tel: +81-11-706-5531

Fax: +81-11-706-7545

E-mail: seino@igm.hokudai.ac.jp

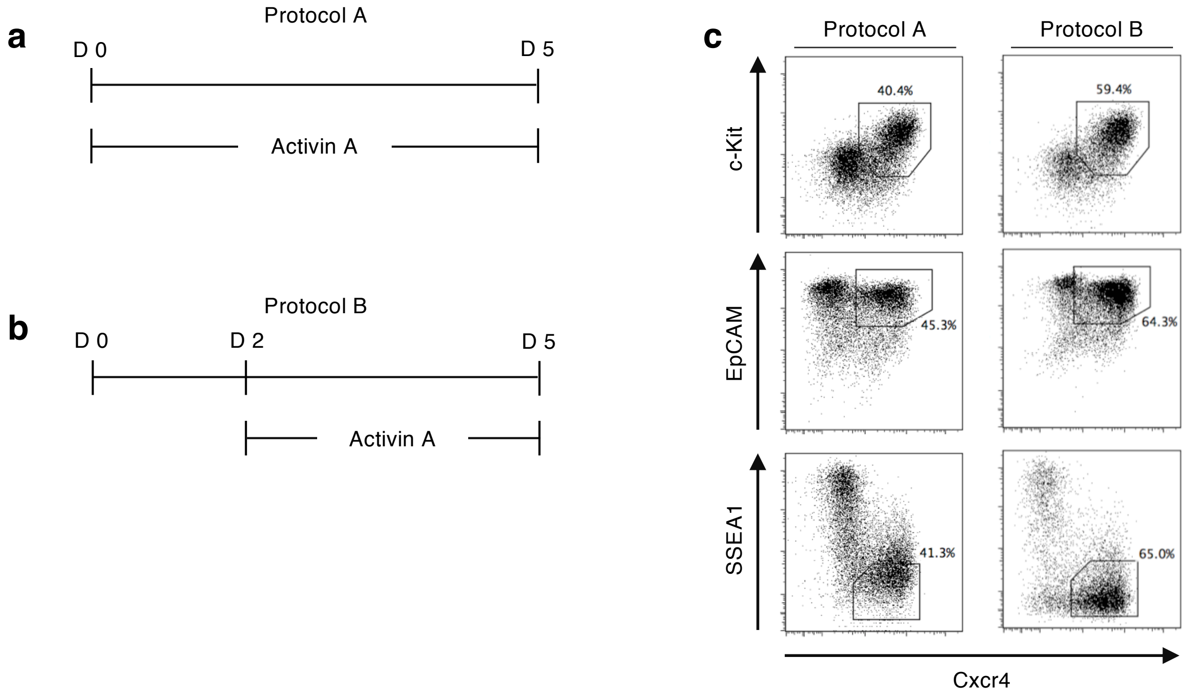

Supplementary figure 1: Definitive endoderm induction with Activin A
(a) (b) DE induction by activin stimulation. Activin A was added to the induction culture from day 0 or day 2. (b) Flow cytometry analysis for DE markers. Cells at day 5 were collected and stained by the indicated antibodies.

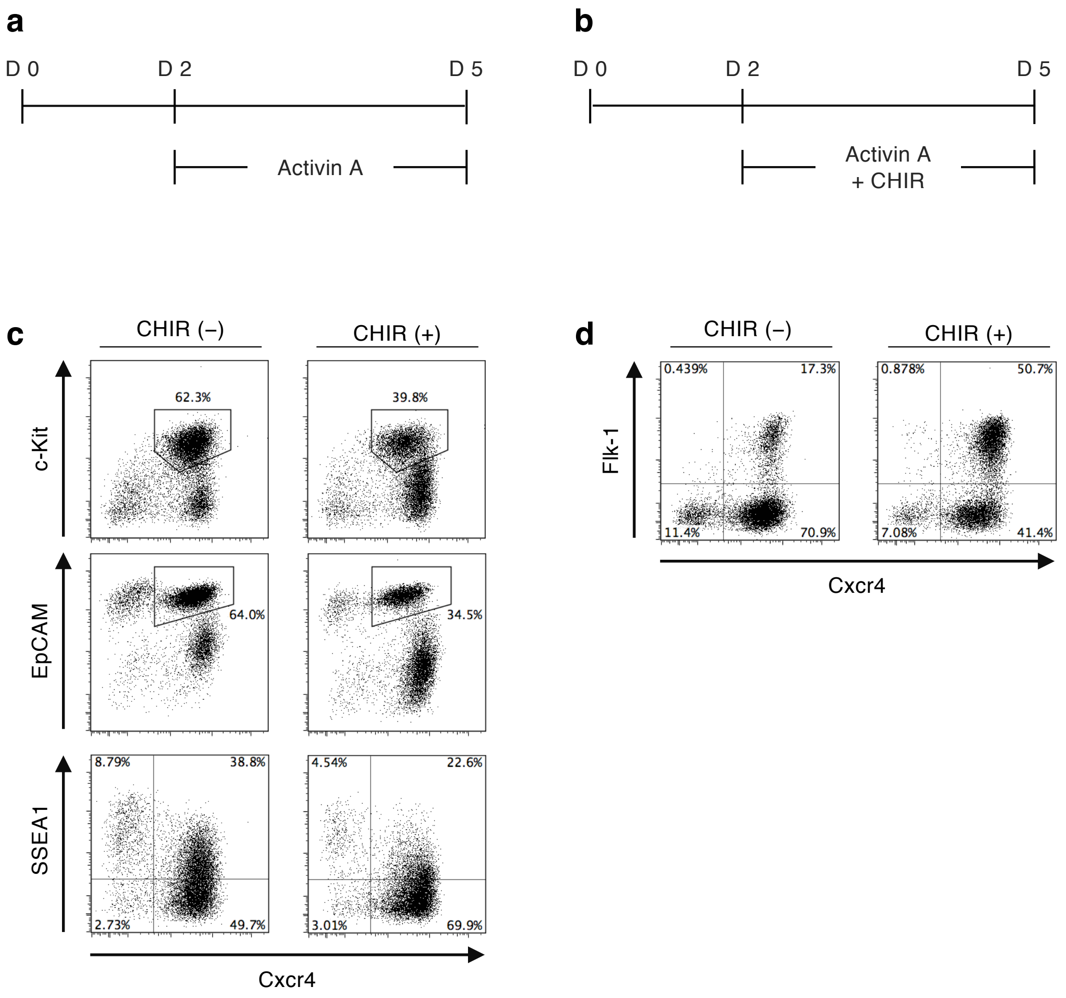

Supplementary figure 2: Wnt/b-catenin signal activation for DE differentiation
(a) (b) DE induction by activin stimulation in the presence or the absence of Wnt/b-catenin signal agonist, CHIR99021. (c) Flow cytometry analysis for DE markers. Wnt signal stimulation resulted in the reduced proportion of residual undifferentiated cells and DE marker positive cells. (d) Analysis for mesodermal cell marker, Flk-1. CHIR increased the number of Flk-1 positive cells.

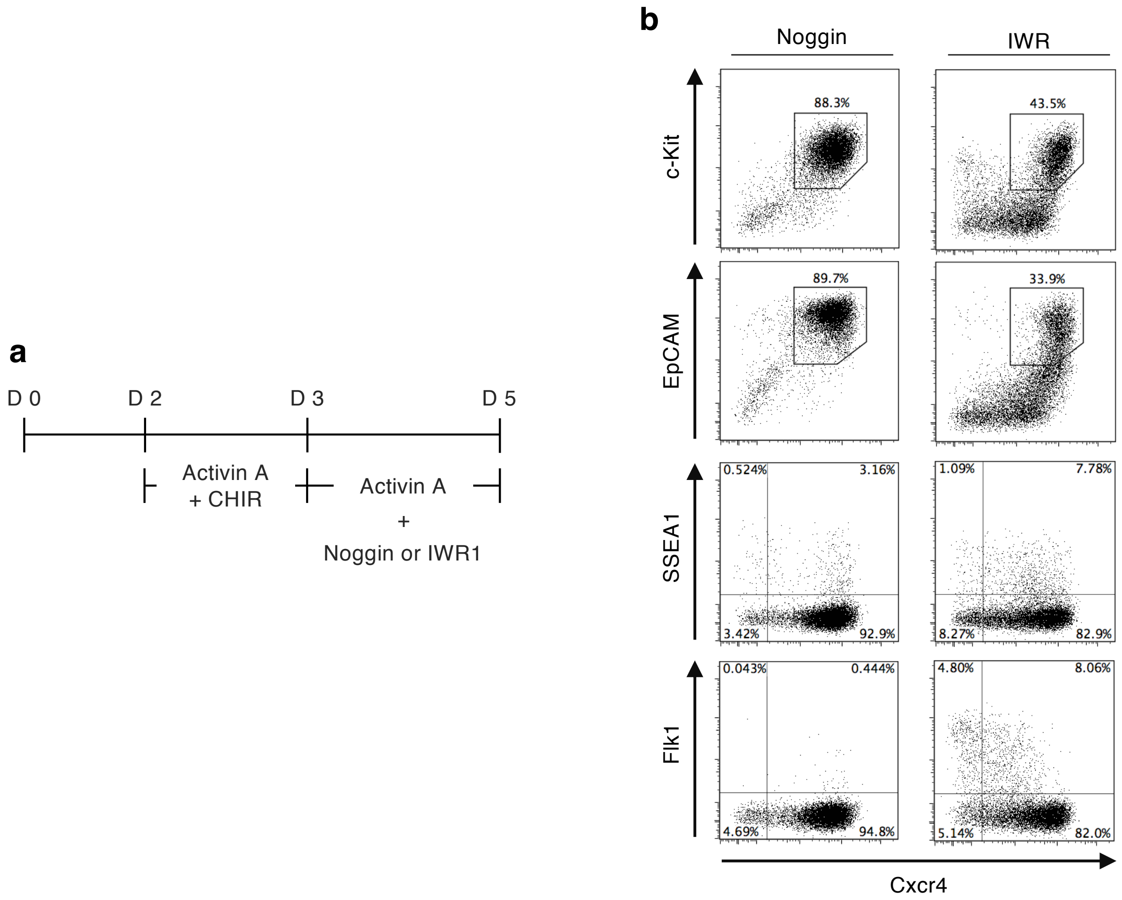

Supplementary figure 3: Inhibition of mesodermal differentiation by BMP antagonist, Noggin, or Wnt antagonist, IWR1.
(a) DE induction by activin stimulation along with the pulse stimulation with CHIR followed by BMP or Wnt signal inhibition.
(c) Flow cytometry analysis for DE markers. Noggin promoted DE differentiation but inhibited mesodermal differentiation.

Supplementary Figure 4

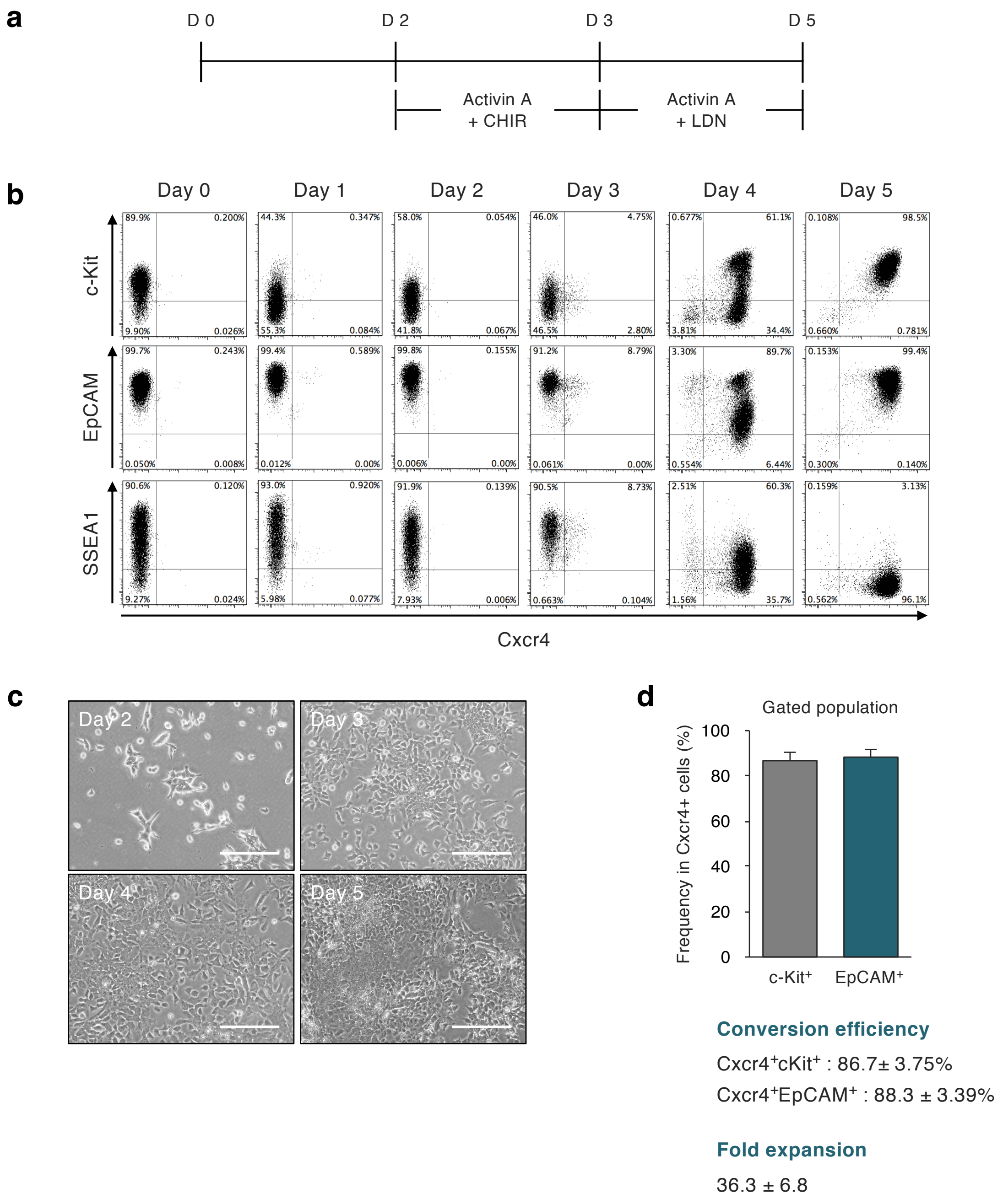

Supplementary figure 4: Detailed conditioning of DE induction by the sequential signal modification.
(a) Addition of LDN193189, BMP signal inhibitor, as a substitute for Noggin. (b) (c) Kinetics of DE marker molecules, and morphological change during DE differentiation. Scale bars describe 100 μm. (d) Mean induction efficiency and fold expansion rate. Error bars describe standard error of the mean.

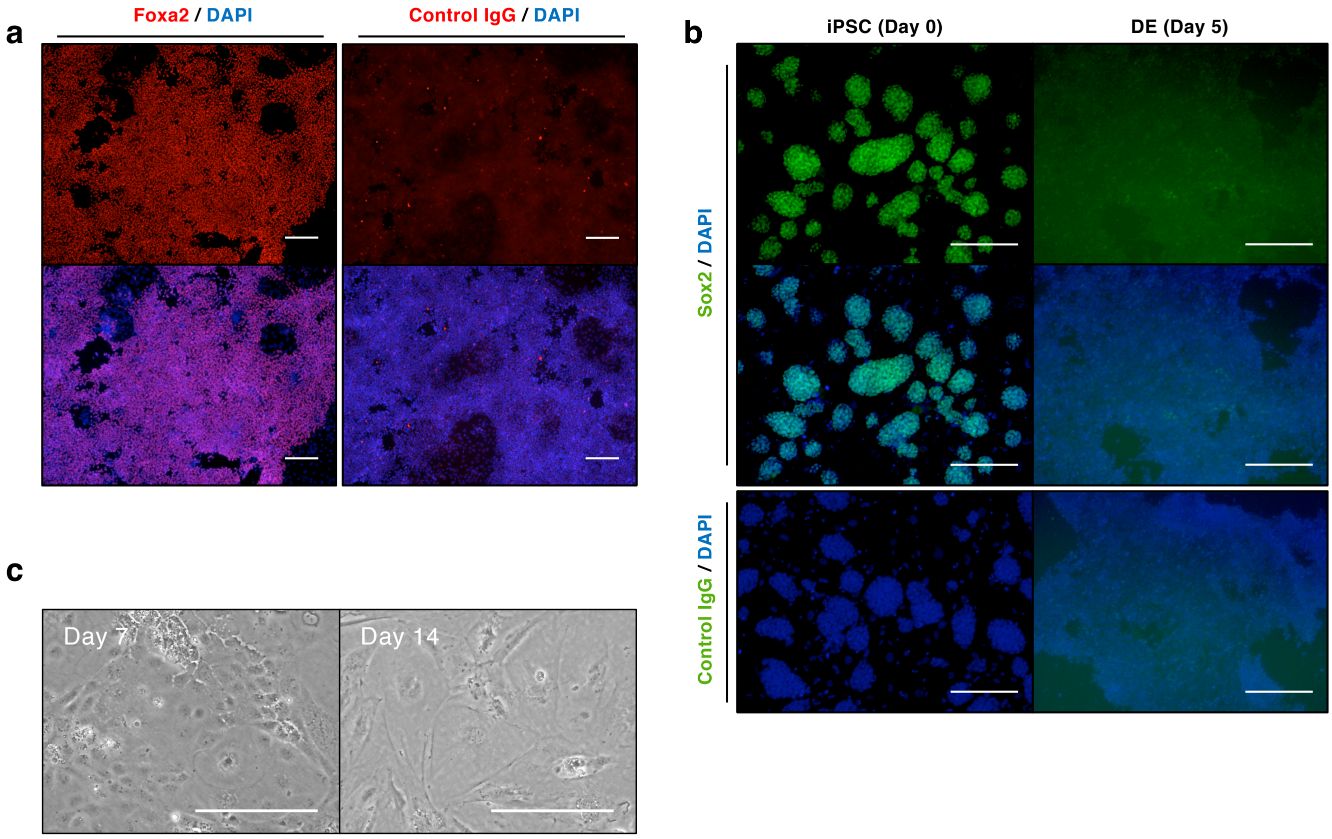

Supplementary figure 5: Foxa2 and Sox2 protein expression analysis in induced definitive endoderm cells and morphology of the cells at day 7 and 14.
(a) Immunostaining for Foxa2 and staining with control IgG. Nuclei were counter stained with DAPI. (b) Immunostaining of iPSC and D5 DE cells for Sox2 and staining with control IgG. Scale bars describe 200 μm. (c) Phase-contrast images of the differentiation cells on day 7 and 14. Scale bars describe 100 μm.

Supplementary Figure 6

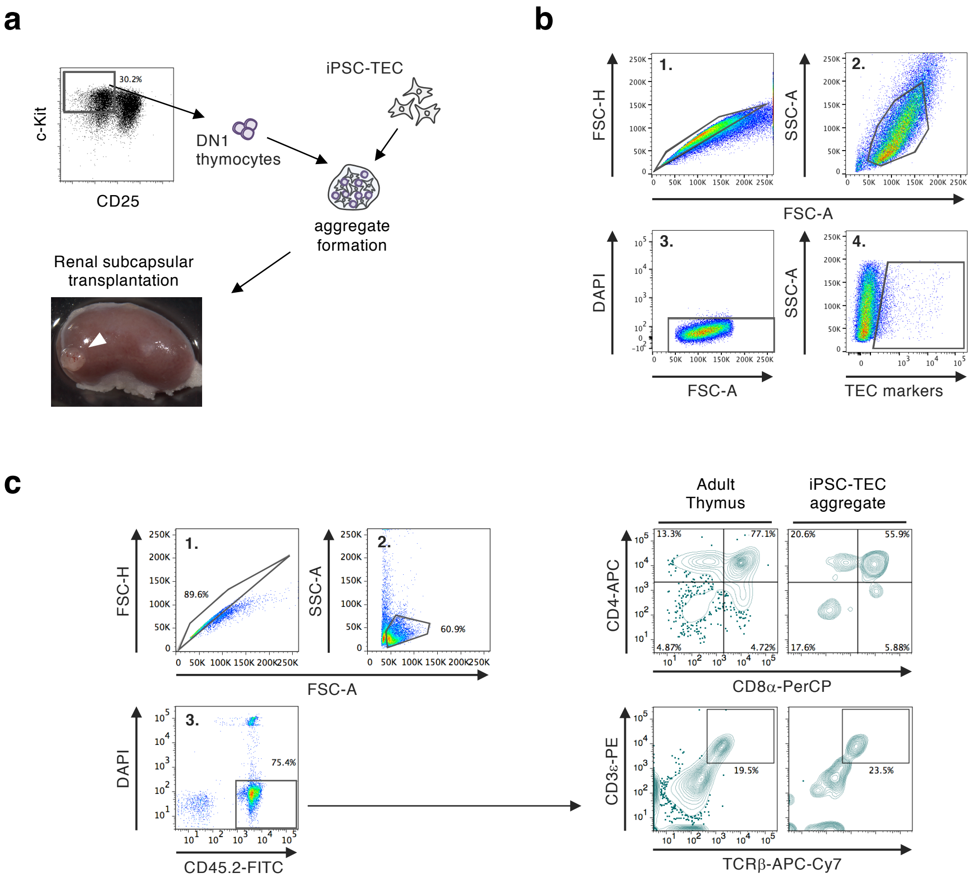

Supplementary figure 6: Transplantation of iPSC-TECs aggregated with DN1 thymocytes.
(a) Schematic overview of aggregate transplantation. Picture shows macroscopic image of transplanted site on day 17 after transplantation. Arrow head indicates recovered aggregate. (b) Plots exemplifying the gating strategy of iPSC-TEC flow cytometry analysis and sorting. TEC markers, EpCAM, Ly51 and UEA-1. (c) Gating strategy for aggregate analysis (left panels) and the results of flow cytometry analysis of recovered aggregates (right panels). The same gating strategy was employed for peripheral blood analysis in Fig. 2d.

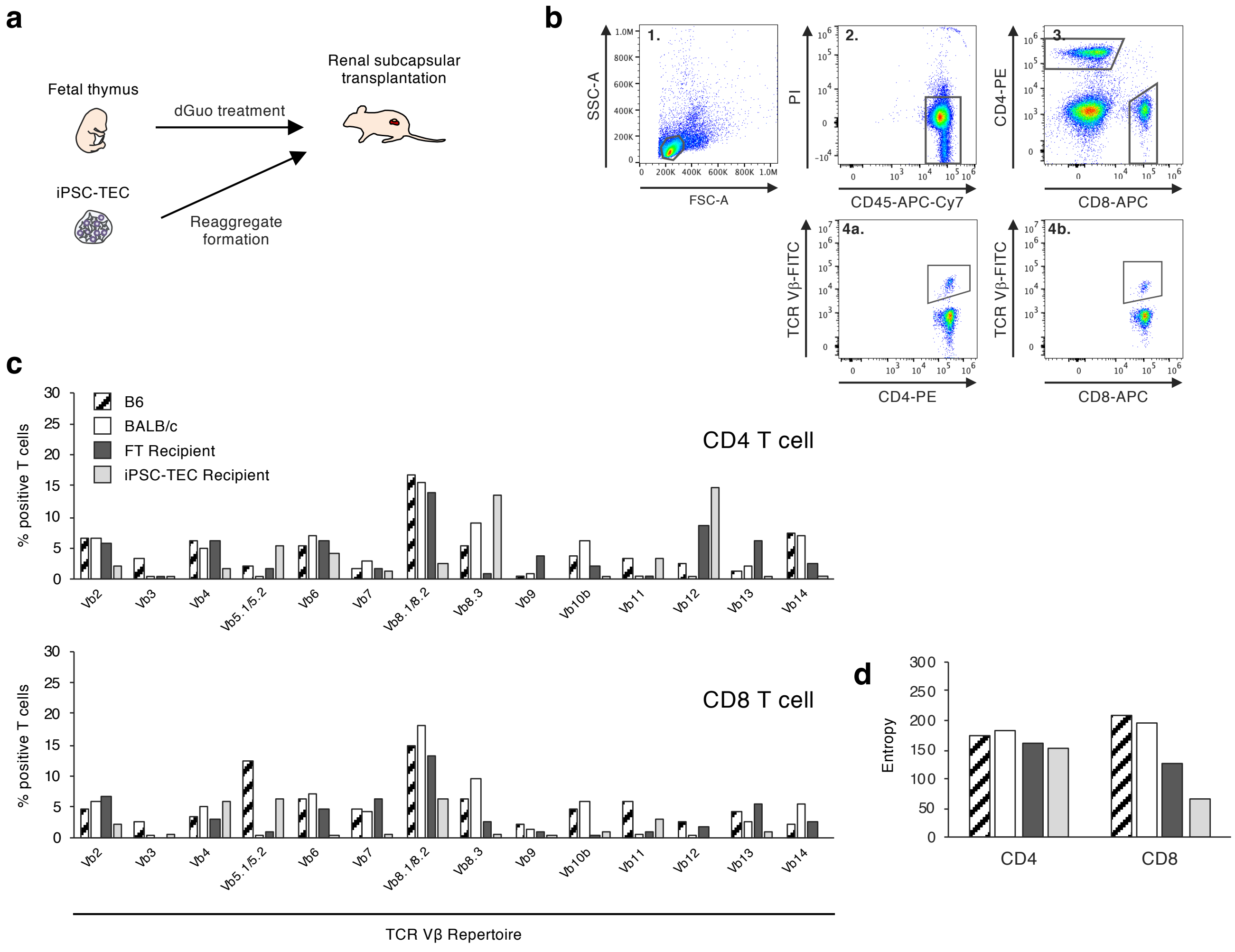

Supplementary figure 7: TCR repertoire diversity analysis after iPSC-TEC transplantation to nude mice.
(a) Schematic overview of repertoire analysis. Deoxyguanosine treated fetal thymus (FT) lobes from E14 B6 fetus or iPSC-TEC aggregates were transplanted to nude mice 4-6 weeks before analysis. (b) Flow cytometry plots exemplifying the gating strategy of TCR repertoire analysis. (c) TCR Vβ analysis of recipient spleen T cells. (d)TCR Vb repertoire entropy analysis in CD4 or CD8 single positive T cells (B6, n=3; BALB/c, n=3; FT recipient, n=3; iPSC-TEC Recipient, n=2; biological replicates).

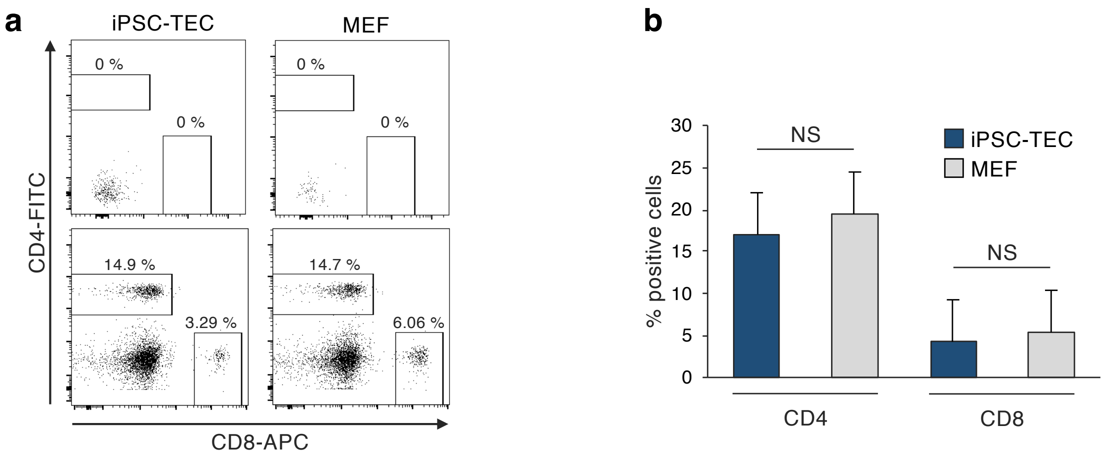

Supplementary figure 8: T cell recovery kinetics of recipient F1 mice.
(a) Plots show representative flow cytometric analysis of recipient mice at the day of (upper) and 4 weeks after (lower) aggregate transplantation. (b) Bar graphs show the frequency of $CD4^+$ or $CD8^+$ cells within live cells (iPSC-TEC, n=6; MEF, n=4). NS, not significant; two-tailed Student's t-test. Error bars describe standard error of the mean.

Supplementary Table1 : Primer list for RT qPCR

Sequences of primers used for RT quantitative PCR

Gene	Forward primer (5'→3')	Reverse primer (5'→3')
Foxa2	CATCCGACTGGAGCAGCTACTA	AGCGCCCACATAGGATGACAT
Sox17	AGAAACTGCAGACCAGAAGCTATCA	GCTCATTGTATCCATGAGGTGACA
Cer1	AGGAGGAAGCCAAGAGGTTC	CATTTGCCAAAGCAAAGGTT
Oct4	CTCGAACCACATCCTTCTCT	GGCGTTCTCTTTGGAAAGGTGTTC
Sox2	GAGTGGAAACTTTTGTCCGAGA	GAAGCGTGTACTTATCCTTCTTCAT
Pax9	TGGAACGTGGGGAACATTCG	GCACGCAGTGAATGGATTGG
Tbx1	CAAGGCTCCGGTGAAGAAGA	TGGAACGTGGGGAACATTCG
Hoxa3	GTCTCTCCCCCTCAAAGTGC	AGATCTTGATCTGGCGCTCG
Foxn1	CTTAAAGGTCAAAGAAGGAAAACACT	GGCTAACAAATAAGTTGGCTGAG
Dll4	GGAAGTACTGTGACCAGCCTAT	CACCATTTCGACAGGGGTTG
Ccl25	TGCAACCTACGTGCTGTGAG	CATCACCATCCTGGGATGACC
Gapdh	TCAAATGGGGTGAGGCCGGT	TTGCTGACAATCTTGAGTGA

Supplementary Table2: Antibody list for flow cytometry and immunofluorescence

Antibody	Conjugation	Clone	Supplier
EpCAM	APC, Biotin	G8.8	Biolegend
Ly51	PE-Cy7, Biotin	6C3	Biolegend
Dll4	APC, Biotin	HMD4-1	Biolegend (APC), Miltenyi (Biotin)
c-Kit	APC	2B8	Biolegend
Cxcr4	PE	L276F12	Biolegend
CD3ε	PE	145-2C11	eBioscience
CD4	FITC, PE	RM4-5	Biolegend
CD8	FITC, PE	53-6.7	eBioscience
CD11b	FITC	M1/70	eBioscience
CD11c	FITC	HL3	BD Pharmingen
CD19	FITC	MB19-1	eBioscience
CD25	PE	3C7	Biolegend
CD45.2	FITC	104	Biolegend
Gr-1	FITC	RB6-8C5	eBioscience
NK1.1	FITC	PK136	eBioscience
TER119	FITC	TER-119	eBioscience
Sox2	None	245610	BD Bioscience
Foxa2	None	Rabbit polyclonal	MERCK
Foxn1	None	Goat polyclonal	Santa Cruz
